# Interleukin-1β is a potential therapeutic target for periodontitis: a narrative review

**DOI:** 10.1038/s41368-019-0068-8

**Published:** 2020-01-02

**Authors:** Ran Cheng, Zhiwu Wu, Mingming Li, Meiying Shao, Tao Hu

**Affiliations:** 1 State Key Laboratory of Oral Diseases & National Clinical Research Center for Oral Diseases & West China Hospital of Stomatology, Sichuan University, Chengdu, China; 2 West China School of Public Health and West China Fourth Hospital, Sichuan University, Chengdu, China

**Keywords:** Periodontitis, Mechanisms of disease, Target identification

## Abstract

Interleukin(IL)-1β, a pro-inflammatory cytokine, was elevated and participates in periodontitis. Not only the link between IL-1β and periodontitis was proved by clinical evidence, but also the increased IL-1β triggers a series of inflammatory reactions and promotes bone resorption. Currently, IL-1β blockage has been therapeutic strategies for autoimmune and autoinflammatory diseases such as rheumatoid arthritis, cryopyrin-associated periodic syndromes, gout and type II diabetes mellitus. It is speculated that IL-1β be a potential therapeutic target for periodontitis. The review focuses on the production, mechanism, present treatments and future potential strategies for IL-1β in periodontitis.

## Clinical evidence regarding interleukin(IL)-1β

Periodontitis is an inflammatory disease that infects tooth supporting tissue and eventually induces tooth loss. At present, the aetiology of periodontitis is characterised by both dysbiosis of the host and oral microbiota.^1^ Various mediators affect the balance in chronic inflammation. The pro-inflammatory cytokines include IL-1β, tumour necrosis factor (TNF)-α, IL-6 and IL-17, which lead to periodontal inflammation and tissue injury. Another group includes regulatory cytokines such as IL-10.^2,3^ IL-1β is a strong stimulator of periodontal tissue destruction. The properties of IL-1β include promoting bone resorption and inducing the production of tissue-degrading proteinases.^4^ In addition to periodontitis, pro-inflammatory cytokines such as TNF-α, IL-1β and IL-6 modulate systemic diseases.^3^ Correspondingly, cytokine-based strategies have the potential to improve both periodontitis and systemic health. This review focuses on new cytokine-based strategies related to IL-1β in periodontitis.

As a pro-inflammatory cytokine, IL-1β participates in inflammation, immune regulation and bone resorption in periodontitis. The clinical findings provide strong evidence about its significance. The biological effects of IL-1β depend on its tissue concentration, which is elevated in periodontitis. Increased levels of IL-1β are frequently detected in the saliva and gingival crevicular fluid (GCF) of patients with periodontitis compared with healthy controls.^5,6^ Patients with deeper pocket depths and more severe bleeding on probing (BOP) have increased levels of GCF IL-1β.^7,8^ Moreover, serum IL-1β in patients with chronic periodontitis also reaches a high level, inducing a systemic effect.^9,10^ This finding demonstrates that IL-1β is a possible causal link between periodontitis and systemic diseases such as cardiovascular diseases.^11^ The nod-like receptor protein-3 (NLRP3) inflammasome signalling pathway, which contributes to the activation of IL-1β, is involved in periodontal pathogenesis. The mRNA expression of the NLRP3 inflammasome complex was enhanced both in gingivitis and in periodontitis.^12^

There are significant increases in the salivary levels of NLRP3, apoptosis-associated speck-like protein containing a caspase recruitment domain (ASC), and IL-1β in chronic periodontitis and aggressive periodontitis.^13^

Additional evidence is that polymorphisms in IL-1 genes might be associated with the risk of periodontitis. Numerous studies have reported that the IL-1β polymorphisms affect the susceptibility to periodontal diseases and their progression. Moreover, the polymorphisms in the IL-1β gene might be associated with increased periodontitis severity.^14^ The studies focus on the polymorphisms leading to transitions between C and T at three positions, 3954/3 953(C → T, rs1143634), −511(C → T, rs16944), and −31(T → C, rs1143627). IL-1β rs1143634 has long been reported as a risk factor in the progression of periodontitis.^15–19^ However, some studies do not support such an association,^20,21^ especially in aggressive periodontitis.^22^ Recent meta-analyses demonstrated that the IL-1β rs1143634 polymorphism is associated with periodontitis.^23,24^ Individuals with IL-1β rs1143634 also show high levels of IL-1β in the GCF,^25^ which adds to the evidence that IL-1β rs1143634 might be a genetic risk factor for periodontitis.

The association between two other polymorphisms and periodontitis is comparably weak. The IL-1β rs16944 polymorphism is associated with susceptibility to periodontitis in the European^20^ and the Chinese population.^26^ However, another report showed no significant association between the IL-1β rs16944 polymorphism and CP susceptibility.^27^ The link between the IL-1β rs1143627 polymorphism and susceptibility to periodontitis is even weaker. The studies show that the IL-1β rs1143627 polymorphism is not related to periodontal disease susceptibility.^18,28^

Clinical evidence verifies that periodontitis leads to excessive IL-1β production locally (GCF and saliva) and systemically (serum). The mechanism of IL-1β production is important for final control.

## The production of IL-1β in periodontitis

IL-1β is mainly expressed by macrophages and dendritic cells. However, gingival fibroblasts, periodontal ligament cells, and osteoblasts can also secrete IL-1β.^29^ The secretion of IL-1β is unique. The cytokine is first produced as a proprotein, which is subsequently proteolyzed into its active form by caspase-1. The inactive precursor, pro-IL-1β, is produced in response to pathogen-associated molecular patterns [PAMPs, e.g., lipopolysaccharide (LPS)] or damage-associated molecular patterns (DAMPs, e.g., HMGB1, ATP). PAMPs and DAMPs function through pattern recognition receptors (PRRs), e.g., Toll-like receptors (TLRs), to activate the signalling adaptor myeloid differentiation primary response 88 (MyD88). MyD88 activation results in the degradation of IκB as a prerequisite for the release of NF-κB dimers and hence promotes pro-IL-1β expression.^30^

Pro-IL-1β is produced as biologically inactive forms that must be proteolytically cleaved to acquire functional activity.^31^ Caspase-1, originally recognised as an IL-1-converting enzyme, is able to cleave pro-IL-1β into active IL-1β, leading to the final secretion. Caspase-1 undergoes activation by a multi-molecular assembly known as the inflammasome. Inflammasomes are composed of the sensor (PRRs including NLRP1, NLRP3, NLRC4, Pyrin and AIM2), the adaptor ASC (apoptosis-associated spec-like protein containing a CARD), and caspase-1.^32,33^ To date, most investigations have focused on the NLRP3 inflammasome, which is involved in various inflammatory diseases.^33–35^ The NLRP3 inflammasome is activated in response to a variety of PAMPs and DAMPs. In macrophages, NLRP3 inflammasome activation is proposed to be a two-signal pathway. The first signal is priming, which is induced by microbial or endogenous stimuli that promote NLRP3 and pro-IL-1β expression through the activation of NF-κB. The second signal is activation, which is triggered by exogenous ATP (eATP), pore-forming toxins and particulate matter sensors, which activate the NLRP3 inflammasome and caspase-1 and cleave pro-IL-1β into its mature form. The protein gasdermin is another substrate of active caspase-1. Active caspase-1 cleaves gasdermin at the N terminus and C terminus. The N terminus of gasdermin forms pores on cell membranes and subsequently induces pyroptosis.^32^

**Fig. 1:**
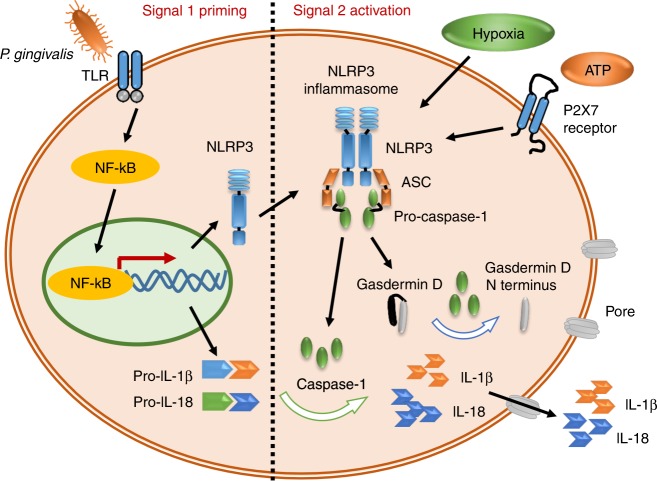
The two signalling pathways for the activation of the NLRP3 inflammasome in periodontitis. Signal 1 is a priming signal. One of pathogen-associated molecular patterns, e.g., *P. gingivalis*, activates the transcriptional factor NF-κB and thus increases the production of NLRP3, pro-IL-1β and pro-IL-18. Signal 2 is an activation signal. One of pathogen-associated molecular patterns, such as ATP, activates the NLRP3 inflammasome via the P2X7 receptor. Hypoxia helps to activate the NLRP3 inflammasome. Activated caspase-1 produces the active form of IL-1β and IL-18. Activated caspase-1 also cleaves gasdermin D at the N terminus, thus forming pores and inducing the pyroptosis of cells.

In recent decades, the production of IL-1β and pyroptotic processes has been studied in the field of periodontitis. The periodontal pathogens belong to PAMPs that initiate IL-1β production. In macrophages, leucocytes and gingival fibroblasts, *Porphyromonas gingivalis* (*P. gingivalis*) is able to activate caspase-1, IL-1β and IL-18. *P. gingivalis* fimbriae, LPS and DNA act as PAMPs and are recognised by several surface and cytosolic PRRs, e.g., TLRs.^36^ However, different cell types vary in their priming and activating pathways. Macrophages^37,38^ and gingival epithelial cells^39^ participate in two signalling pathways. They first promote NLRP3 and pro-IL-1β expression in response to *P. gingivalis*. Subsequently, the binding of eATP to the P2X7 receptor, an ion-gated channel, is required for the maturation of IL-1β and final secretion.^40^ However, human monocytes require only one signal for secreting IL-1β after stimulation with TLR2 or TLR4 receptors due to the endogenous release of ATP and the activation of the P2X7 receptor.^41^ Human gingival fibroblasts are special in their response to *P. gingivalis* for the maturation of IL-1β. Supragingival biofilms could enhance caspase 1 activation and the expression of IL-1β and IL-18 in gingival fibroblasts.^42^ However, subgingival biofilms, including *P. gingivalis*, downregulate NLRP3 and IL-1β.^43^ It was suggested that hypoxic conditions were essential in the maturation of IL-1β.^44^ The diagram is shown in Fig. 1.

*Actinobacillus actinomycetemcomitans* (*A. actinomycetemcomitans*), the pathogen of aggressive periodontitis, upregulates IL-1β expression in human mononuclear leucocytes^45^ and macrophages.^46^ Leukotoxin, an important virulence factor that targets leucocytes, rapidly activates caspase-1 and thus induces a massive secretion of IL-1β in human monocytes and macrophages.^47,48^ The NLRP3^45,49^ and AIM2 inflammasomes,^50^ reactive oxygen species and cathepsin B^51^ might be involved in the process. However, the exact signalling pathways are not clear and require further investigation.

## The role of IL-1β in periodontitis

**Fig. 2:**
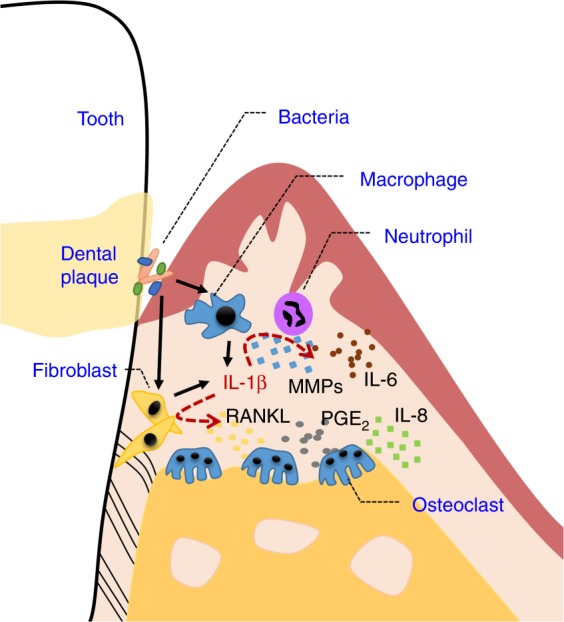
The role of IL-1β in periodontitis. IL-1β promotes the secretion of MMPs, RANKL, PGE_2_, IL-6, IL-8, etc., which promotes osteoclastogenesis.

After secretion, the accumulated IL-1β triggers a series of inflammatory reactions and participates in the pathology of periodontitis.^52^ Generally, IL-1β in the inflammatory site accounts for increased local blood flow, leucocyte recruitment and neutrophil infiltration.^53^ Furthermore, IL-1β acts as a strong stimulator of bone resorption, making it a featured cytokine in periodontitis. The major mechanisms are shown in Fig. 2.

IL-1β increases the expression of collagenolytic enzymes, matrix metalloproteinases (MMPs), which contribute to extracellular matrix degradation and in turn lead to bone resorption and tissue destruction.^53,54^ MMP-9 is an important indicator of the severity and progression of periodontitis.^55–58^ IL-1β upregulates MMP-9 expression in various cell types involved in periodontal inflammation, including osteoblasts, osteoclasts, neutrophils, and cementoblasts.^59,60^ In addition, IL-1β stimulated the production of other MMPs, e.g., MMP-1 and/or MMP-3, in human periodontal ligament cells and gingival fibroblast cells.^61–63^

The receptor activator of nuclear factor kappa-B ligand (RANKL) and osteoprotegerin (OPG) system regulates osteoclastogenesis. Increased RANKL and decreased OPG is a feature of periodontitis.^64^ IL-1β upregulates RANKL and thus stimulates osteoclastogenesis.^65^

RANKL is a member of the membrane-associated TNF ligand superfamily and is detected on osteoblasts, bone marrow stromal cells, activated T cells, periodontal ligament fibroblasts, cementoblasts and endothelial cells.^66–69^

RANKL binds its receptor, receptor activator of NF-κB (RANK), expressed on the osteoclast precursor cells, resulting in the activation of the osteoclast.^70^ The binding of RANKL to RANK finally activates the transcription of NFATc1, c-Fos and NF-κB-related genes, which are upregulated in periodontitis^71,72^ and modulate the proliferation, differentiation and activation of osteoclasts.^73,74^ IL-1β also regulates the production of OPG, which is a soluble receptor for RANKL that prevents it from binding to RANK.^75,76^ The RANK/RANKL/OPG system is influenced by IL-1β and has been associated with bone metabolism.^77^

Other than these direct effects, some indirect effects are influenced by IL-1β. IL-1β increases PGE_2_ synthesis in fibroblast cells.^61,75^ PGE_2_ induces the expression of RANKL as well.^78^ IL-1β increases the expression of CX3CL1, which mediates the migration of osteoclast precursors under the osteoblast layer and finally leads to osteoclastogenesis.^79^ In response to IL-1β, the fibroblasts in the periodontium produce pro-inflammatory mediators, e.g., IL-6, IL-8,^80–82^ which are reported to stimulate bone resorption.^83^

In brief, IL-1β has a long-lasting effect on osteoclastogenesis, which leads to bone resorption.^65,67^

## The influence of current therapies on IL-1β

The treatments for periodontitis consist of scaling and root planing (SRP), surgery and some adjunctive therapies, e.g., antibiotics, laser therapy or antimicrobial photodynamic therapy (aPDT).^84^ The therapies not only improve clinical parameters but also influence the levels of cytokines. However, conventional SRP and surgery are not efficient in reducing the IL-1 levels. Some adjunctive therapies have unexpected effects.

### SRP and surgery

SRP is a cost-effective, minimally invasive and non-surgical treatment to prevent and/or control periodontal diseases. However, the mechanical treatment may be incomplete in eliminating the pathogenic microorganisms.^85,86^ The levels of pro-inflammatory cytokines may be continuously high after treatment. Previous studies found that SRP did not significantly reduce the IL-1 levels.^87,88^ Some recent studies also show that the IL-1β level is decreased after SRP treatment.^89,90^ SRP results in a decrease in the level of IL-1β in GCF or saliva. However, the amounts are still higher than in the healthy group.^91,92^ Furthermore, an obvious increase in the IL-1β level is detected even after papillary flap debridement, which suggests that trauma and wound healing result in prolonged production of IL-1β.^93^

Due to the difficult access by SRP, the removal of plaque and infectious cells can be impaired in some sites. Although SRP treatment is effective in reducing clinical parameters, it is insufficient in the anti-inflammatory treatment for increased mediators. Thus, some adjuvant therapies have been analysed for this purpose.

### Antibiotics

The local and systemic administration of antibiotics is an adjunctive treatment for periodontitis. Drugs such as minocycline, doxycycline, roxithromycin, amoxicillin and metronidazole are typically used.^94^ Clinical studies have shown that antibiotics promote clinical parameters. However, its effect on IL-1β is limited.

The local administration of minocycline microspheres in periodontal pockets during periodontal maintenance significantly reduces IL-1β in GCF at 6 months.^94^ However, there are no changes over 24 months.^95^ The systemic administration of amoxicillin and metronidazole is an adjunctive treatment for aggressive periodontitis. This treatment reduces the IL-1β level in GCF at 3 months,^96^ but it has no effect at the 6-month follow-up.^97,98^ Roxithromycin therapy is applied in cyclosporine-A-induced gingival overgrowth. However, there is no effect on the level of IL-1β.^99^ Furthermore, antibiotics at or below minimal inhibitory concentrations increase IL-1β secretion in macrophages by inducing the shedding of LPS by *P. gingivalis*.^100^

### Laser therapy

Lasers could be used as a monotherapy, as an adjunct to SRP, or in a surgery for incision. Laser therapy provided minimally invasive soft tissue and biostimulatory effects on tissue regeneration.^101,102^ Laser therapy significantly improved clinical parameters, including the plaque index, gingival index, probing depth, clinical attachment level and BOP.^89^ The biochemical parameters IL-1β and TNF-α were reduced as well.^103^

For the treatment of periodontal inflammation, Nd:YAG therapy combined with SRP was more effective than SRP alone in reducing GCF IL-1β levels at the 1-week, 3-month and 6-month follow-ups.^104,105^ Nd:YAG laser therapy was also effective in reducing serum IL-1β in patients with periodontal diseases.^106^ Er,Cr:YSGG laser therapy decreased IL-1β in both aggressive periodontitis and chronic periodontitis.^107^ However, SRP plus the adjunctive use of diode laser therapy did not reduce GCF IL-1β levels compared to SRP alone,^108^ nor did it reduce inflammation in sites with ≥5 mm PD.^109^ Er:YAG laser irradiation had little effect on GCF IL-1β levels, both in the SRP+laser group and in the laser-only group.^110^

Different types of lasers are more effective in some specific clinical areas. For example, the Nd:YAG laser could be used to remove gingival tissue and was the only laser for possible periodontal regeneration.^111^ The present evidence showed that the Nd:YAG laser was also effective in reducing IL-1β. However, more evidence about the types of lasers, different wavelengths and the best exposure protocol is needed to assess laser therapy.

### Antimicrobial photodynamic therapy (aPDT)

aPDT is an antimicrobial treatment. The therapy is an oxygen-dependent photochemical reaction that occurs upon light-triggered activation of a certain photosensitising compound. The reaction finally leads to the production of cytotoxic reactive oxygen species, predominantly singlet oxygen, which kills microorganisms including viruses, bacteria and fungi.^112^ Due to its antibacterial effects, aPDT along with SRP is advantageous and effective in the treatment of periodontitis.^113^ aPDT plus SRP resulted in a significant reduction in probing depth and a suppression of IL-1β and MMP-8 in GCF when compared with SRP alone^114^ aPDT plus SRP also decreased the amount of periodontal pathogens (red and orange complexes) and lowered the IL-1β/ IL-10 ratio in GCF compared to SRP alone.^115^ Compared with the systemic use of metronidazole, aPDT significantly lowered the bacterial load and IL-1β level of the gingival sulcus of rats with periodontitis.^116^ These results suggest that aPDT has an anti-inflammatory effect on IL-1β.

Overall, conventional treatment, including SRP, surgery and antibiotics, has limited effects on IL-1β. Some new adjunctive treatments, including laser therapy and antimicrobial photodynamic therapy, are comparably more efficient than conventional treatment. Some potential adjunctive treatments might be new strategies to target IL-1β.

## Therapeutic strategies for the potential use of IL-1 blockage in periodontitis

Other than the current therapies for IL-1β, are there any potential strategies to target IL-1β in periodontitis?

The pathological role of IL-1 is being discovered in a broadening list of diseases in which blocking strategies of IL-1 could be effective. The diseases include all classic autoimmune diseases and autoinflammatory diseases. The classic autoinflammatory diseases are rare, including neonatal-onset multisystem inflammatory disease, Muckle-Wells syndrome, familial cold-induced autoinflammatory syndrome, hyperimmunoglobulin D syndrome, etc. Recently, the benefit of IL-1 blockage has expanded to rare conditions, such as gout, diabetes mellitus and even myeloma. These conditions share the common feature that IL-1 is involved in their pathogenesis.^117^ Urate crystals promote IL-1β secretion and thus induce joint inflammation in gout.^118^ Type II diabetes mellitus is a chronic inflammatory disease in which β cells are continuously destroyed by IL-1^119^ and is improved by treatment with an IL-1 receptor antagonist.^120^ IL-1β produced by myeloma cells stimulatesim the secretion of IL-6 by adjacent stromal cells. The excess IL-6 in turn promotes the proliferation of the pre-myeloma cells.^121^

The effect of IL-1β in periodontitis is discussed above. Studies early in 1998 showed that blocking IL-1β resulted in reduced progression of periodontal bone loss and attachment loss in a non-human primate model of periodontitis,^122,123^ suggesting that IL-1 blockage is potentially effective in periodontitis. With the current development of IL-1 blocking agents, the strategy of blocking IL-1β may have new prospects.

**Fig. 3:**
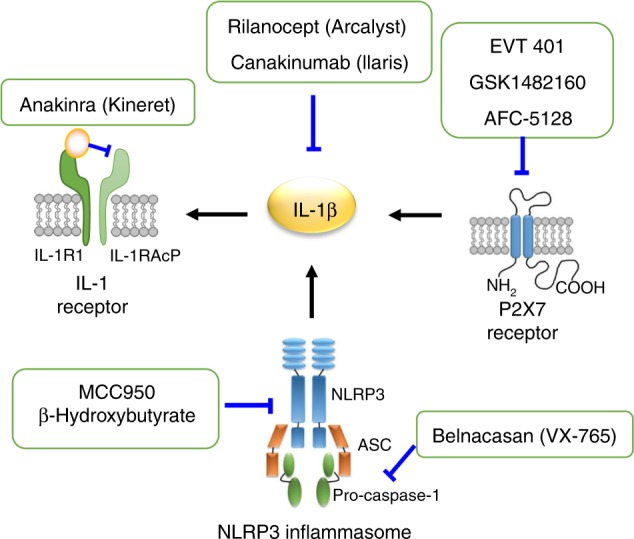
The agents that block IL-1β activity, including IL-1β antibodies, IL-1 receptor antagonist, NLRP3 inflammasome inhibitors and P2X7 antagonists. IL-1R1, subunit of IL-1 receptor; IL-1RAcP, IL-1R accessory protein.

Since the first blocking agent, anakinra (Kineret; Amgen) was introduced in 1993,^124^ a variety of agents have been produced for IL-1 blockage, including IL-1β/receptor antagonists, inflammasome inhibitors and P2X7 antagonists. These antagonists/antibodies are shown in Fig. 3.

### IL-1β/receptor antagonists/antibodies

#### Anakinra (Kineret)

Anakinra is a recombinant homologue of the IL-1 receptor antagonist. It competes with IL-1α and IL-1β to bind to the IL-1 receptor and thus reduces the activity. Anakinra was first approved in the US in 2001 and in Europe in 2001 for the treatment of rheumatoid arthritis. It has been proven to be effective in a variety of inflammatory diseases.^124,125^ In 2018, the National Health Service (England) published a Clinical Commissioning Policy for anakinra to treat periodic fevers and autoinflammatory diseases at all ages. However, anakinra has a short half-life of 4–6 h; therefore, daily subcutaneous injections were required.^126^

#### Rilonacept (Arcalyst)

Rilonacept is a recombinant IL-1 antagonist consisting of the extracellular portion of the human IL-1 receptor and the IL-1 receptor accessory protein fused with the Fc portion of human IgG1. The extracellular portion has a strong affinity for both IL-1α and IL-1β, thereby neutralising their activities and functioning as an “IL-1 trap”.^127,128^ It was approved by the FDA in 2008 for the treatment of cryopyrin-associated periodic syndromes (CAPS). Presently, a number of clinical trials are being developed for chronic inflammatory diseases, including type I diabetes (NCT00962026), atherosclerosis (NCT00417417), hepatitis (NCT01903798) and chronic kidney disease (NCT01663103).^126^ Furthermore, rilonacept has a longer half-life of 6–8 days; therefore, the interval of injections can be extended to a week.^129^

#### Canakinumab (Ilaris)

Canakinumab is a high-affinity, fully human monoclonal anti-IL-1β antibody designed to exclusively bind and neutralise human IL-1β. Canakinumab was approved by the FDA in 2009 for the treatment of CAPS. Some more clinical trials for diseases such as osteoarthritis (NCT01160822), chronic obstructive pulmonary disease (NCT00581945), type II diabetes (NCT00605475), atherosclerosis (NCT00995930) and rheumatoid arthritis (NCT00504595, NCT00424346) are undergoing. The half-life of canakinumab is as long as 26 days, which ensures that the injection can be administered bimonthly, a substantial advantage over anakinra and rilonacept.^126^

### Inflammasome inhibitors

The NLRP3 inflammasome contributes to the maturation of IL-1β. The inflammasome inhibitors include the targets caspase-1 and NLRP3.

#### Caspase 1 inhibitor

Belnacasan (VX-765, Vertex Pharmaceuticals) is a selective inhibitor of caspase-1. It is an orally absorbed prodrug that is converted to VRT-043198 under the action of plasma and liver esterase.^129^ Currently, Belnacasan is used to suppress caspase-1 activity in inflammatory diseases. It inhibits collagen-induced arthritis and lung inflammation in mouse models.^130,131^ It partly decreases bone resorption in the periapical lesion of rat experimental apical periodontitis.^80^ In addition to its anti-inflammatory effect, VX-765 also has a protective role against cell death. It combines with a P2Y12 antagonist, cangrelor, to reduce myocardial infarct size and preserve ventricular function.^132^ VX-765 is well tolerated in phase II clinical trials in patients with epilepsy and^133^ also possesses neuroprotective activity in multiple system atrophy in a mouse model.^134^

#### NLRP3 inflammasome activation inhibitors

MCC950 and β-hydroxybutyrate are two small-molecule inhibitors of the NLRP3 inflammasome. MCC950 is a diarylsulfonylurea-containing compound that selectively inhibits the activation of the NLRP3 inflammasome. MCC950 acts specifically on the NLRP3 inflammasome but not on NLRP1, AIM2 or NLRC4 inflammasomes. MCC950 blocks ASC oligomerization and the activation of caspase-1 and IL-1β in mouse and human macrophages.^135^ Thus, MCC950 reduces IL-1β production and may serve as a potential target for inflammatory diseases^136–138^ It also provides protection against injury. MCC950 significantly reduces the development of atherosclerotic lesions, cardiac infarction and brain injury after intracerebral haemorrhage in mice.^139–141^

β-Hydroxybutyrate (BHB) is a ketone body that is produced as an adaptive starvation response during a negative energy balance.^142^ BHB inhibits the NLRP3 inflammasome in macrophages and neutrophils.^143,144^ Although IL-1β or IL-1 receptor antagonists have been approved by the FDA and have been shown to be effective in some clinical trials, the high costs and potential risk may limit their use. BHB, an endogenous ketone, may function as a regulatory metabolite to regulate inflammation. It has an anti-inflammatory effect on retinal damage.^145^ BHB prevents NLRP3 inflammasome activation in both mouse and human neutrophils and reduces urate-crystal-induced inflammation in individuals with gout.^144^

Compared with the currently used IL-1β or IL-1 receptor antagonists, MCC950 and BHB are less expensive to produce. Due to their specificity, MCC950 and BHB will benefit NLRP3-targeted therapies for both acute and chronic inflammatory diseases.^146^

### P2X7 antagonists

The P2X7 receptor is an ionotropic ATP-gated cation channel. DAMPs, such as eATP, which is released from damaged cells, activate the purinergic receptor and function as a second signal for assembly of the NLRP3 inflammasome. The NLRP3 inflammasome in turn initiates and amplifies the innate immune and IL-1β-dependent pro-inflammatory responses.^147,148^ The P2X7 receptor antagonist has potential in the treatment of chronic pain, inflammation and cancer.^149,150^ The P2X7 receptor antagonists oATP and A-740003 inhibit eATP-induced IL-1β secretion in *P. gingivalis*-infected macrophages.^38^ An antagonist of the P2X7 receptor, AZ106006120, reduces neutrophil infiltration and the secretion of pro-inflammatory cytokines in a mouse model of acute lung injury.^151^ Several P2X7 antagonists are in clinical trials for inflammatory or pain-related diseases. Unfortunately, AstraZeneca’s AZD-9056 and Pfizer’s CE-224,535 failed in phase II clinical trials. Evotec’s EVT 401 is in phase II clinical trials. GSK’s GSK1482160 and Affectis Pharmaceutical’s AFC-5128 are currently in phase I clinical trials.^149^

## Other potential agents that influence IL-1β in periodontitis

Some in vitro and in vivo experiments showed that some other agents have anti-inflammatory properties against IL-1β. They might be candidates for future adjuvant treatments.

### Plant-derived substances

Plant-derived substances are largely utilised in immunomodulatory therapy. Some of them have anti-inflammatory effects that modulate the host response in inflammation.^152^ Resveratrol and curcumin are produced by several plants and belong to polyphenols. They are capable of reducing IL-1β and bone loss in animal models of experimental periodontitis.^152^ The anti-inflammatory properties of curcumin are similar to those of chlorhexidine-metronidazole.^153^ Curcumin attenuates the production of IL-1β and TNF-α stimulated by LPS in rat gingival fibroblasts in vitro.^154^ Piperine isolated from black and long peppers exhibits anti-inflammatory activity. It inhibits alveolar bone loss and downregulates the expression of IL-1β in rat periodontitis.^155^ Plumbagin is extracted from the roots of *Plumbago zeylanica* L. It significantly decreases the expression of TNF-α, IL-1β and IL-6 and decelerates bone destruction in rats with chronic periodontitis.^156^

Compared to the systemic antimicrobial agents or the chemical agent chlorhexidine gluconate, plant-derived substances partly avoid the problems of drug resistance, overdoses and a number of adverse effects.^157,158^ Plant-derived substances have great potential as adjuvant therapy for periodontal diseases.

### Anti-inflammatory agents

Some anti-inflammatory or antioxidant agents are beneficial for reducing IL-1β. Metformin is an agent for the treatment of type II diabetes. Metformin activates AMP-activated protein kinase, which has been shown to exert significant anti-inflammatory and immunosuppressive effects.^159,160^ Metformin reduces the concentrations of IL-1β and bone loss in a rat model of experimental periodontitis.^161^ In vitro, metformin inhibits LPS-influenced IL-1β production in human gingival fibroblast cells.^162^

Vitamin E, a potent antioxidant, is important to the host’s antioxidant defence and immune functions.^163^ Vitamin E decreases the secretion of IL-1β in human gingival fibroblasts stimulated with *P. gingivalis* LPS. As a result, vitamin E may have anti-inflammatory effects against *P. gingivalis*.^164^

### Antibodies or antagonists

There are also some antibodies or antagonists that indirectly influence IL-1β. Infliximab is a monoclonal antibody against TNF-α. It reduces the expression of IL-1β in gingiva and has significant anti-inflammatory and bone-protective effects in Wistar rats with experimental periodontitis.^165^ Bortezomib, a proteasome inhibitor, is used as an anticancer drug. Bortezomib interrupts the breaking-down process of the proteasome and promotes the death of cancer cells. The anticancer activity is accompanied by an anti-inflammatory effect. It has been reported that bortezomib inhibits the expression of IL-1β and prevents alveolar bone absorption in experimental periodontitis.^166^

## Conclusion

IL-1β is an important pro-inflammatory cytokine and participates in periodontitis. As a strong stimulator of bone resorption, continuous bone loss may be induced by IL-1β. Conventional therapies, SRP, surgery and antibiotics have limited effects on IL-1β. IL-1β blockage by receptor antagonists, antibodies, inhibitors, plant-derived substances and anti-inflammatory agents is beneficial for reducing IL-1β. More investigation is necessary for IL-1β blockage to be used in periodontal treatment or as an adjunctive treatment in the future.
